# Case report: specific phobia of vaginal penetration in a pregnant patient

**DOI:** 10.3389/fpsyt.2023.1218900

**Published:** 2023-08-01

**Authors:** Wenqi Geng, Jinya Cao, Li Jin, Jing Wei

**Affiliations:** ^1^Department of Psychological Medicine, Peking Union Medical College Hospital, Chinese Academy of Medical Sciences and Peking Union Medical College, Beijing, China; ^2^National Clinical Research Center for Obstetric and Gynecologic Diseases, Department of Obstetrics and Gynecology, Peking Union Medical College Hospital, Chinese Academy of Medical Sciences and Peking Union Medical College, Beijing, China

**Keywords:** phobic disorders, prenatal care, referral and consultation, psychopharmacology, muscle relaxation

## Abstract

Specific phobia is frequently unrecognized or untreated unless it causes significant impairment. In this report, we documented a rare case of a pregnant patient who had a specific fear related to vaginal penetration. Due to abnormal fetal cardiac development in the second trimester, the patient was admitted for termination of pregnancy. The patient’s persistent request for surgical termination via cesarean delivery prompted the obstetrician to seek psychiatric consultation for tokophobia, a labor- and childbirth-related phobia. The consulting psychiatrist discovered that the patient had developed a significant fear of vaginal penetration during adolescence. Throughout the extended period of this specific phobia, the patient established a range of avoidance strategies. Had it not been for the unforeseen need for abortion, her phobia may not have been identified. Psychoeducation on specific phobias, exposure therapy, muscle relaxation techniques, and the administration of anxiolytics were implemented. The pregnancy was terminated through a vaginal labor induction procedure 2 days later. Collaboration across disciplines is necessary to support a thorough assessment of obstetric patients who express hesitancy toward vaginal delivery.

## 1. Introduction

The physiological processes of gestation and parturition, which are exclusive to the female, can lead to a variety of outcomes and reactions for individuals. Some women experience feelings of elation and eager anticipation with regards to pregnancy, while others may experience fear and a desire to avoid the situation (1–3). It was estimated that 14% of females experienced tokophobia, which is a clinical term used to describe a pathological fear of pregnancy and/or childbirth (2). Patients with tokophobia frequently opt for caesarean section to avoid the vaginal birth process (4, 5). In this report, we present the diagnosis and treatment of a patient initially presenting with tokophobia who consistently resisted vaginal induction, but was eventually diagnosed with a specific phobia of vaginal penetration. A combination of cognitive-behavioral therapy, progressive muscle relaxation, and anxiolytics was implemented for the management of her specific phobia, effectively facilitating labor induction in this individual. This is the first case report of a pregnant patient with a specific fear of vaginal penetration.

## 2. Case presentation

### 2.1. Patient information

The patient, who was in her late thirties, was referred by an obstetrician to receive psychiatric consultation in her second trimester for an evaluation concerning symptoms of “fear of vaginal induction.” Previously during the second trimester prenatal examination, abnormal fetal cardiac development was observed. Consequently, the patient was hospitalized for pregnancy termination. Based on the fetus’ gestational age, dilation and evacuation was deemed inappropriate, and a cesarean section was determined to be the most viable surgical option. However, as the established criteria for a surgical abortion had not been met, and in order to minimize potential complications such as hemorrhage and infection while maximizing future fertility prospects, a labor induction through vaginal delivery was recommended instead. Despite this recommendation for labor induction through vaginal delivery, the patient expressed a strong preference for a caesarean section. The obstetrician observed the patient’s distress during their discussion and suspected the presence of tokophobia. As the conversation progressed, the patient expressed conflicting emotions regarding the procedure, stating, “I want to terminate the pregnancy as soon as possible. Ideally, I’d like to pursue a natural method [vaginal delivery], but I’m also reluctant to go through the childbirth process.” Subsequently, she agreed to undergo psychiatric evaluation and receive appropriate intervention.

This was the patient’s initial pregnancy during her 10-year marriage, and the patient denied any issues within her marital relationship. The patient had a stable job position. The patient was diagnosed with myocarditis during adolescence, but achieved a complete recovery following appropriate treatment. She had a family history of diabetes and hypertension but did not have a family history of mental disorders.

### 2.2. Clinical findings

After reviewing the patient’s medical records, the consulting psychiatrist noticed that the patient consistently declined to undergo pelvic examinations. The psychiatric consultant visited the patient in her hospital room, accompanied by her spouse. The patient’s vital signs were stable and the physical examination was unremarkable. She was alert and well-oriented, cooperating with the mental status examination. She had no symptoms of chronic depression or anxiety. She had a complete understanding of the obstetrician’s medical recommendations and the advantages and potential disadvantages of both cesarean delivery and vaginal induction. When asked about her prior choice of a cesarean section and concerns regarding giving birth, she had difficulty providing an explanation. She also seemed uneasy when questioned about the possibility of vaginal induction. She denied fear of labor pains. Following a comprehensive and empathetic inquiry, she revealed that she experienced a significant fear of vaginal penetration, which arose during her adolescence. While it was difficult to pinpoint the exact nature of the “ultimate fear,” it persisted into her adulthood. She had no recollection of experiencing any physical or psychological trauma. Due to the fear of vaginal penetration, the patient exhibited various avoidant behaviors. These included opting for sanitary pads instead of tampons during menstruation, constantly declining pelvic examinations and skipping annual gynecological check-ups, engaging in only a limited number of vaginal intercourses over the course of a 10-year marriage, and repeatedly postponing attempts at pregnancy. She explicitly declined vaginal procedures including pelvic examinations during this hospitalization. She stated, “When the nurses tried to perform pelvic examinations, my fear was so strong, I would instinctively kick them out.” “I can’t imagine or tolerate any thing getting in or out of my vagina.” She denied fear of other specific environments or objects. The patient struggled with natural conception and experienced a profound sense of relief upon confirmation of pregnancy. However, she was also anxious about the forthcoming delivery process, as there was a possibility of vaginal delivery. Hence, she had consistently made arrangements for a cesarean delivery. Upon discovering that the fetus had significant developmental abnormalities, the patient elected to proceed with termination of pregnancy. After a brief period of grief, the patient expressed anxiety about vaginal delivery and requested a cesarean delivery to terminate the pregnancy. She did not perceive any possible solutions to alleviate her fear, such as medication or psychotherapy, at that time. There was no evidence of impaired cognitive ability, psychosis, manic or depressive episodes, obsessive thoughts, or compulsive behaviors.

### 2.3. Diagnostic assessment

Laboratory blood tests, including complete hemogram, comprehensive metabolic panel, reproductive hormones and thyroid function, yielded results within the normal range. The patient denied any substance use. There were no identifiable factors that could account for the patient’s phobia. According to the Diagnostic and Statistical Manual of Mental Disorders-Fifth Edition (DSM-5) criteria as shown in Table 1 (6), she was diagnosed with a specific phobia, other type. Based on the patient’s long-term pattern of avoidance behaviors, it can be inferred that her fear is not restricted to the process of childbirth, but rather to the specific situation of having foreign objects (including fetus) entering her vagina. Likewise, the patient’s specific phobia impacts her sexual behavior, resulting in a considerably low frequency of vaginal intercourse. Additionally, there was no evidence of sexual dysfunction, pelvic discomfort or vaginismus. Therefore, the patient should not be diagnosed with tokophobia. Instead, a diagnosis of specific phobia of vaginal penetration would be more appropriate.

**TABLE 1:** Diagnostic criteria for specific phobia in Diagnostic and Statistical Manual of Mental Disorders-Fifth Edition (DSM-5).

1	Marked fear or anxiety about a specific object or situation (e.g., flying, heights, animals, receiving an injection, seeing blood).
2	The phobic object or situation almost always provokes immediate fear or anxiety.
3	The phobic object or situation is actively avoided or endured with intense fear or anxiety.
4	The fear or anxiety is out of proportion to the actual danger posed by the specific object or situation and to the sociocultural context.
5	The fear, anxiety, or avoidance is persistent, typically lasting for 6 months or more.
6	The fear, anxiety, or avoidance causes clinically significant distress or impairment in social, occupational, or other important areas of functioning.
7	The disturbance is not better explained by the symptoms of another mental disorder, including fear, anxiety, and avoidance of situations associated with panic-like symptoms or other incapacitating symptoms (as in agoraphobia): objects or situations related to obsessions (as in obsessive-compulsive disorder); reminders of traumatic events (as in posttraumatic stress disorder); separation from home or attachment figures (as in separation anxiety disorder); or social situations (as in social anxiety disorder).

### 2.4. Therapeutic intervention

Based on the patient’s request for a prompt termination of pregnancy, a limited set of interventions was discussed. Following the presentation of diagnostic results and treatment options, the patient agreed to short-term psychotherapy, medication, and relaxation techniques prior to vaginal delivery. The intended outcome of these interventions was to mitigate fear and enable tolerance for vaginal induction. If the patient proved unable to tolerate vaginal delivery, a caesarean section would be performed to facilitate termination of pregnancy. Given that epidural analgesia was not regularly administered in this facility, it was suggested that spinal analgesia be employed in order to alleviate pain and assist the patient’s phobia. However, the patient did not agree with this recommendation as she felt it was not necessary. As the patient could not recall any significant past traumas, the primary approach proposed was cognitive behavioral therapy. Psychoeducation on specific phobia, exposure to medically-necessary procedures involving mild to moderate vaginal penetration, progressive muscle relaxation techniques, and the implementation of anxiolytic medication were utilized. The diagnosis and treatment were also explained to obstetricians. The patients were administered lorazepam at a dosage of 0.5 mg three times daily. She engaged in three daily sessions of progressive muscle relaxation, with each session lasting 20 min. During each session, the patient was directed to sequentially contract and relax muscle groups throughout the body, commencing with the head, followed by the neck and shoulders, upper extremities, abdomen, back, and concluding with the lower extremities, while incorporating deep breathing techniques. Two days later, the patient deemed herself mentally prepared for a vaginal delivery. Despite her fears, she was determined to proceed with the abortion without further delay. Consequently, the patient underwent labor induction and delivered the fetus vaginally. Along with labor pain, the patient reported experiencing heart palpitations and muscle tension in her upper extremities, though she managed it well overall. Subsequently, the dosage of lorazepam was gradually tapered to 0.5 mg every night.

### 2.5. Follow-up and outcomes

**FIGURE 1:**
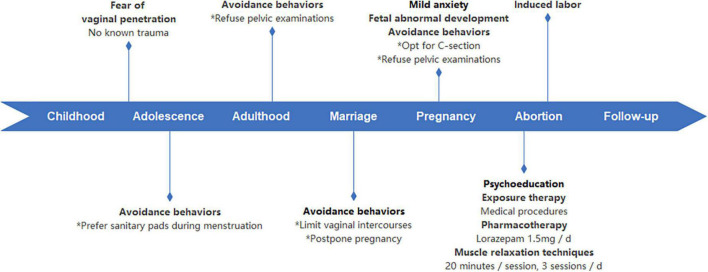
Timeline of case.

One-month post-discharge, the patient underwent a single telephone follow-up with the consulting psychiatrist. She reported a gradual improvement in her physical state. Although she experienced sadness when reminded of her abortion, she did not exhibit symptoms of depression in her daily mood. The patient discontinued lorazepam medication. The patient reported experiencing mild levels of anxiety and discomfort during the labor process. Despite this, she did not describe the experience as traumatic. Furthermore, she did not exhibit symptoms of intrusive thoughts, nocturnal disturbances, or heightened vigilance. The patient highly appreciated the benefits of psychiatric consultation, stating: “I had always felt ashamed of my issue and avoided discussing it with others. Acknowledging it and learning how to overcome my phobia has given me a lot of courage.” While the patient still experiences a degree of vaginal penetration phobia, she has noticed a decrease in severity. Additionally, she expressed her intent to resume psychotherapy, in preparation for any future pregnancies, but declined to participate in psychotherapy at that time, stating: “My phobia is currently not my primary concern.” The patient also gave consent for the publication of this report. The timeline of this case is summarized in Figure 1.

## 3. Discussion

Our report documents a unique case of a female patient presenting with a specific phobia of vaginal penetration that remained undetected until a labor induction through vaginal delivery was recommended during her pregnancy. Presenting as tokophobia, or fear of childbirth, the patient’s fear of vaginal childbirth was clarified to be specifically related to a phobia of vaginal penetration. This particular phobia has been reported infrequently. Vonk and Thyer (7) conducted a case study in 1995 involving a 25-year-old female patient with a fear of vaginal penetration. The patient was unmarried and voluntarily sought help for her fear that was affecting her ability to experience intimacy with partners. Graduated exposure therapy helped the patient to overcome her phobia, and she was eventually able to have vaginal intercourse. In our case, the patient was already pregnant, rendering the initial recognition of a concealed diagnosis of specific phobia considerably more challenging. Despite reaching the goal of completing vaginal delivery after a short-term multifaceted therapy, follow-up results showed that the phobia was not cured, and the patient was hesitant to seek further help at that time.

### 3.1. Diagnosis and differential diagnosis of specific phobia

Specific phobic disorders are frequently unrecognized or untreated unless they cause significant impairment. A multinational epidemiological survey revealed that the lifetime prevalence of specific phobia was greater in high and upper-middle income nations compared to low and lower-middle income nations (8). For instance, the lifetime prevalence in the United States was 12.5%, whereas that in China was 2.6% (8). The actual prevalence may exceed the reported figures owing to the fact that only a minority of patients with specific phobia actively seek help, and they also tend to wait longer before seeking help comparing to those with other anxiety disorders (9–11). The reason for this reluctance may stem from the fact that specific phobia results in restricted social impairment for an individual, which can account for its relatively low level of disability when compared to other disorders (12, 13).

Our report illustrates a typical case of specific phobia. Prior to the diagnosis, the patient had been experiencing a fear of vaginal penetration for over two decades, and to a certain extent, she and her spouse had adjusted their lifestyle accordingly. In a hypothetical scenario where the patient was not recommended to undergo an unexpected labor induction, it is possible that the pregnancy would have resulted in a caesarean delivery, and as a consequence, the patient’s specific phobia would not have been addressed in a clinical setting.

Comorbidity of physical illnesses and mental disorders is frequently observed among patients in general hospitals (14), and failure to identify or manage comorbid psychiatric conditions can result in extended hospitalization periods, increased medical expenses, and suboptimal outcomes of medical illnesses (15–17). This case report highlights how mental conditions can impede routine medical care, and potentially compromise adherence to standard of care. In clinical practice, non-psychiatric healthcare professionals may identify phobias through patient non-adherence to, distress associated with, or avoidance of diagnosis and/or treatment. For example, the patient in this case reported avoiding vaginal procedures, while others may avoid undergoing CT or MR tests due to claustrophobia. Furthermore, intense anxiety or fear that arises from exposure to a specific scene or event can serve as key diagnostic markers. Kannappan and Middleman (18) described a case that presented with medical complications, specifically dehydration, attributed to emetophobia following an episode of viral gastroenteritis. The patient’s profound anxiety was the primary cause of a marked decline in quality of life and went unrecognized by healthcare professionals until a thorough medical assessment had taken place. Raising awareness and recognition of mental and psychological disorders among non-psychiatric healthcare personnel will facilitate prompt consultation and enhance the overall wellbeing of patients. In the meantime, interdisciplinary cooperation can facilitate comprehensive management of the patient’s psychosomatic condition.

The differential diagnosis of specific phobias can present its challenges. Sexual trauma often leads to post-traumatic stress disorder (PTSD), and physicians should consider diagnosing PTSD if the patient’s fear is associated with a previous trauma or triggers related to trauma, accompanied by symptoms including flashbacks, heightened vigilance, and nightmares. While the patient in this case exhibited a particular fear focused on vaginal penetration, there was no clear indication of a traumatic experience in her history. In the case reported by Vonk and Thyer (7), the patient mentioned during treatment that her mother had recalled an accident involving a genital tear while the patient was playing in the playground as a child. However, she had no memory of the event nor did she experience flashbacks or heightened vigilance to suggest PTSD. People experiencing obsessive-compulsive disorder (OCD) may avoid specific objects or situations to prevent compulsive behavior. For example, individuals who feel compelled to clean may avoid public restrooms to prevent the compulsion for cleaning after exposure. However, the patient in this case denied obsessions and compulsions, and there was no clear history of obsessions or compulsions concerning for OCD in her history. It is also essential to distinguish avoidance behavior related to social interaction from social phobia disorder. While both social phobia and specific phobia are considered phobic anxiety disorders, the fear in social phobia is directed specifically toward social interactions and potential scrutiny by others.

### 3.2. Treatment of specific phobia

The psychological treatment of specific phobia involves behavioral therapy, cognitive therapy, hypnotherapy, pharmacotherapy, and additional modalities (19–21). In fact, specific phobia is considered one of the most treatable disorders (22, 23). However, different approaches exhibit varied efficacy across subgroups of specific phobia (19). Most phobias respond well to *in vivo* exposure therapy and systematic desensitization, with the former associated with higher dropout rates. Cognitive therapy and applied muscle tension techniques have shown efficacy in the treatment of claustrophobia and blood-injury phobia, respectively (19). Progressive muscle relaxation (PMR) involves instructing patients to tense and subsequently release targeted muscle groups as a means of self-relaxation, frequently integrated with exposure therapy. Several studies have demonstrated the effectiveness of PMR in treating specific phobias (24, 25). Regarding the formulation of treatment, more therapy sessions are indicative of more positive treatment outcomes (20). Wright et al. (26) reported that a one session treatment demonstrated comparable clinical efficacy both immediately after treatment and during a 6-month follow-up, as compared to a multi-session cognitive behavioral therapy, in the treatment of specific phobias among children and young people. There have been reports of utilizing escitalopram in combination with perospirone to treat sophophobia, and another study employed aripiprazole to treat phagophobia (27, 28). The patient of our study encountered a considerably constrained condition in contrast to previously documented cases. Additionally, the time frame for phobia intervention was also limited. We therefore opted for a combination of anxiolytics along with PMR as the acute phase treatment regimen to attain a prompt response. Although the pelvic floor muscles were not the primary focus of our PMR intervention, it is plausible that the relaxation effects are distributed uniformly throughout the body, potentially including the pelvic floor muscles. This could yield positive outcomes for the patient. The patient successfully completed the labor induction through vagina delivery. A 1-month post-discharge follow-up indicated persistence of the specific phobia. Further therapy would be indicated for treatment of the specific phobia; however, there appeared to be some reluctance from the patient. Research has revealed that around 25% of patients may exhibit hesitancy in undergoing exposure therapy owing to overwhelming phobia-related anxiety (29). Another plausible explanation is that, similar to the patient who had years of “effective” avoidance experience in our study, a significant proportion of individuals with phobias refrain from seeking medical intervention as they are capable of evading the triggering stimuli (20). It is important to note that certain patients may experience sexual phobias stemming from inadequate education, particularly in certain cultural contexts (30). As such, health education should be integrated in cognitive therapy in such circumstances. For sex-related phobias, prior research has indicated that involving sexual partners in the treatment process can yield favorable outcomes (7, 30).

### 3.3. Conclusion

Not all patients presenting with tokophobia, or more specifically fear of vaginal delivery, are fearful of pain or obstetric complications as healthcare professionals might typically assume. Here we present a rare case of specific phobia of vaginal penetration. Obstetric providers should consider psychiatric consultation when the patient exhibits significant emotional distress or behaviors concerning for avoidance that could impact their care, as effective collaboration between obstetricians and psychiatrists is crucial for timely recognition and proper management of complex cases with similar presentations.

## Data availability statement

The original contributions presented in this study are included in this article/supplementary material, further inquiries can be directed to the corresponding authors.

## Ethics statement

The studies involving human participants were reviewed and approved by The Ethical Committee of Peking Union Medical College Hospital. The patients/participants provided their written informed consent to participate in this study. Written informed consent was obtained from the participant/patient(s) for the publication of this case report.

## Author contributions

JC and WG: conceptualization and data curation. WG: writing–original draft. WG, JC, LJ, and JW: writing–review and editing. JW: supervision. All authors contributed to the article and approved the submitted version.
